# What Occupation Neuroses Really Are*Reprinted from the Transactions of the Fifteenth International Congress on Hygiene and Demography.

**Published:** 1914-04

**Authors:** Tom A. Williams

**Affiliations:** M. B., C. M. (Edinburgh), Washington, D. C.


For Texas Medical Journal.

### What Occupation Neuroses Really Are.*

BY DR. TOM A. WILLIAMS, M. B., C. M. (EDINBURGH), WASHINGTON, D. C.

The term "neurosis," where it concerns occupational disabilities, is a misnomer, for disorders of occupation to which this name has been given are in reality psychodynamic inhibitions or disorders in the habitual series of co-ordinated associations gained by education in some art. A want of harmony in the controlling of the mechanism is the fault. Hence the disharmony is always psychological.

This is easily proved by the fact that the neuromuscular apparatus which fails to perform a particular occupational act can quite well accomplish any other act, and this by means of the same muscles, nerves, and brain areas. Hence, the interpretations of Hartenberg that hypotonia is the efficient cause of the professional dyskinesias attributed to neurosis fall to the ground. Nor can we accept Kouindjy's explanation of an ataxia, which he believes because of the favorable effects of re-education.

Hypotonia or ataxia, when present, are mere secondary effects of a primitive dyskinesia induced by a disharmony which has originated ideationally, although the mechanism through which it occurs is largely emotional. The following considerations are explanatory:

The induction of emotions by ideas is more clearly shown in a more acute form than occurs during the steady pursuit of an occupation. We find it best illustrated in the psychogenetic effects of accidents, and the maintenance of these by the patient's own painful ruminations thereupon, through what amounts to chronic fear and its effects upon the internal secretions.

### THE ROLE OF EMOTION.

An accident short of lesion may create in a susceptible person so great a fear as to cause a sudden increase of secretion by the

*Reprinted from the Transactions of the Fifteenth International Congress on Hygiene and Demography.

thyroid gland. The recent researches of Crile have shown that this occurs almost constantly in patients with hyperthyroidism when they are frightened by the prospects of an operation: it occurs in these subjects as a result of anxiety or unusual excitement. Crile believes that the thyroid gland is an organ by means of which there is rapidly available a store of an activating substance for the use of the neuromuscular apparatus when there is special need of its greatest power. Such an occasion is presented by the need for escape from danger. As the preservation of the species in locomotory animals may depend upon capacity to respond suddenly with the maximum of vigor against impending danger, there has developed through the long course of phylogeny this special organ of storing and rapidly setting free, when required, such a substance as the thyroid juice. But it would be a mistake to confine the need for the greater activation of the gland to physical escape from danger in the crude sense. In the higher animals, life is no longer regulated by experiences purely phylogenetic by the instincts. It is in the main controlled by ontogenetic incidents which we call experience, and which modify the reactions in fashions incalculably complex. The determinators of these modifications we call association of ideas. Now, quite apart from the fear of bodily harm, there is a vast series of possible events which man seeks to avoid, and which he apprehends as dangers from which to escape. It is reasonable to believe that psychological situations of this kind are capable of reflexly demanding the hyperactivation afforded by the thyroid juice. That is, fear from any source may create a temporary hyperthyroidism.

But that the thyroid secretion is not the only one modified by emotion has recently been shown by a brilliant research of Cannon. He has shown that the emotion of fear in animals is capable of stimulating the flow of adrenal secretion. He demonstrated that, in frightened animals, the blood from the adrenal vein is so rich in adrenal substance as to be capable of inhibiting peristalsis in an isolated strip of intestinal muscle. This is due to the presence of the adrenal substance in appreciable amount, since it requires contact of the latter (with the intestinal strip) in a one-millionth solution at least to inhibit peristalsis.

We knew that the emotion of fear could inhibit gastric secretion, and Pawlow has shown that certain emotions of anticipatory joy can induce a flow of this secretion.

While it lasts, the fear state presents marked physical symptoms. It does so through the intervention of the autonomic nervous system, which cannot be controlled directly by volition, except in rare cases, and those only after much practice. One such case I saw in Philadelphia recently with Dr. J. Madison Taylor. This man, an athlete, had devoted much attention to control of his reactions. He is able to provoke at will the pilomotor reflex, which produces the gooseskin appearance. He claims, too, that he can modify the rate of his pulse, but he did not succeed in demonstrating this clearly to me. He is able also to bring tears to his eyes by purely psychological means. Careful analysis shows that none of these reactions occur from pure willing. To produce them he has to assume a peculiar emotional state, which he describes as one of mystery. His introspection of this is not clear enough for one to say that it is not a feeling of horror. He thinks it is not, because it is rather pleasant, but the pleasure may be that of accomplishing something for which he strives. The analysis need not further detain us, for I quote the case only to draw attention to the impossibility of affecting the autonomic nervous system by direct volition, and to show the need of the intermediary of emotion for provoking autonomic reactions.

The above case may be compared with the simpler one described by Babinski, and which I observed in Paris in 1907. This timorous young girl, without practice in control, was so apprehensive of the pin scratch used to elicit the plantar reflex that she involuntarily drew up foot and leg at the approach of the pin and then occurred pilomotor contraction upon the skin over the upper and outer part of the thigh overlying the muscles (tensor fasciæ latæ reflex), which contract in the defense reaction when one strokes the sole. The patient could not control this response in any way; and its strict localization is unlike that of the preceding case, in whom the goose skin was general when it came at all.

Of course, these cases are somewhat peculiar, but horripilation is a very common reaction to alarm.

Other common consequences are alteration of pulse rate and pressure. The frequency may become more or less than normal and the pressure may be raised or lowered. Perhaps these differences depend upon the neurological type, or they may be functions of the varying responsiveness of the ductless glands in various individuals or at different times.

Upon the digestion, the effects of alarm are well known to be malappetite and constipation with all their accompaniments.

Upon the respiration, fear acts by a complete arrest followed by exaltation, or mere shallowing may ensue. It is a consequence of the inhibition of muscular activity known as fear paralysis.

This may be regarded as a phylogenetic mechanism for stabilizing an individual preparatory to efficient action.

The effect of terror upon the flow of urine and control of the bladder needs only mention.

Even the ancients knew, too, how fear arrested the sexual functions.

But the autonomic modifications of secretions soon cease as the fear shock of the accident fades; and, in a few days, at most, the animal experimented upon or the human being insulted resumes stable equilibrium.

PSYCHOGENETIC FACTORS IN MODIFYING IDEAS, FEELINGS, AND ACTS.

*Sinistrosis.*

But this benign eventually is often interfered with in human beings by the property they possess of reviving in memory the ideas which clothe situations with horror, apprehension, anxiety. Especially prone to this damaging sequence are persons whose imagination has been made rampant by the cultivation of the credulous fears of childhood; their fear reaction to that which they do not understand is a dominant one, and they are easily beset by an idea linked with fear. The commonest of the fears which result from accident or injury is that of bodily harm. It is very hard for a person of this type, when ignorant of his own structure and functions, to shake off the foreboding created bv an impressive catastrophe, and it must not be forgotten that what others regard as trifling, the victim may look upon as catastrophic when judged by its possible effect on him.

Prepossession by the idea of one's own disability is an inevitable consequence. This leads to abstraction from and inattention to the affairs of ordinary life, which, if not trifling by comparison in the patient's mind, at least cannot claim the attention properly needed. Hence ensues the well known diminution of the capacity to think, work, or take part in social life. This incapacity, when the patient becomes aware of it, leads him to still further accentuate the result of his injury, and thus to augment his alarm about his health. Thus is constituted the vicious circle of hypochondria. Even a nosophobia may ensue, such as the fear of lost manhood, insanity, paralysis. Alarm at this impending disaster must, of course, be distinguished from the primary alarm due to the accident itself.

The next step in the drama is the reaction against the actual absence of physical signs of injury, and the reassurances of medical men. This takes the form of an unconscious search by the patient of ratification for his belief that he is indeed damaged. Hence arises the familiar exaggerations and falsifications of symptoms. These are made in perfect good faith and honest belief, but they lead to the stimulation of disease pictures previously in mind, or acquired in the course of the disorder.

It is only after the patient begins to be convinced in his heart that he is mistaken that there ensues the deliberate self-deception of the desperate effort to preserve the respect of himself and his friends which he feels he would lose by admitting the absence of physical disorder after all.

By means of this mechanism may arise what Brissaud called sinistrosis. Even a favorable settlement of a lawsuit may not remove this attitude. Only skillful psyclotherapy will do so; and, in severe cases, considerable time must be allowed to wear down the sinister habit of mind.

This mechanism was clearly illustrated in the case of sinistrosis, which I fully reported to the International Congress of Industrial Accidents at Rome, in 1909. The rapidity of the success of the treatment in this and other cases is due to the thoroughness of the analysis which leads to the comprehension as there described. The treatment consists of rational psychomotor discipline by which the patient is enabled to re-educate himself and get rid of a dyskinesis, which is, in reality, a dysboulia.

### THE MECHANISM IN OCCUPATIONAL NEUROSES.

In the occupational psychoses, for so they are, the mechanism is entirely similar, as is well seen in the case which follows:

F. W., aged 30, a telegraph operator, complained of cramping of the wrist and hand while using the Morse sender, the typewriter, or the pen. Cramp sometimes occurs upon lifting a cup to drink, but only if he thinks of it. It occurs also upon brushing his teeth, but only those on the right side. It does not occur upon lifting up articles, unless he uses the fingers alone, when cramp may occur, but he can wind his watch, button his coat and collar, and perform other ordinary acts without cramping.

*The Cramps.*—The movements consist of a flexing of the wrist when using the Morse key; an extension of the ulnar fingers when using the typewriter; a rigidity of the wrist when brushing his teeth; a giving way and radial rotation of the wrist on lifting a cup to his mouth; a rigidity of the whole arm when attempting to write.

He is completely incapacitated from his usual work, but is able to address envelopes in pencil in the rigid fashion mentioned.

*The History.*—The personal and family history were unimportant, except that he had a nervous sister and was disappointed that he could not follow his father's profession of medicine, and had to work from boyhood. He neither drank nor smoked. Although ambitious, he does not think that telegraphing was his forte, for, though he had tried hard enough, others with less education did better. He had not realized this, however, until he was too old to easily find something else which paid as well. Perhaps he had found learning harder than most people, because he tried to learn at night, while working as a clerk in the daytime.

Although ten years ago, after falling from a bicycle and dislocating his shoulder, he had worked in pain for five weeks before reduction was made (not, however, at the key, but in writing); no dibability had followed. He had not learned the Morse key in the usual way of the lads who pick it up as office boys and evolve into operators, for he began intentionally to learn when he entered an office at 14, and after four years could send forty to forty-five words per minute steadily, with few mistakes.

*Origin of the Cramp.*—The cramp occurred suddenly six years age with "a kind of loss of use of the wrist, which would go up in the air while he was using the keys." At the same time the hand would flex. At the period when this occurred he had been sending in the daytime for a stockbroker from 9:30 a. m. to 4 p. m., whereas formerly he had never used the key for more than three or four hours, and that in the evenings. The stockbroker's work had to be done quickly, and he felt it a strain, but he liked it in spite of its arduousness, for it did not seem to interfere with his health, although he had to eat lunch in the few minutes he could snatch at his desk. His only worry, before the cramp occurred, was the fear of not being able to do his work and of losing his position. Just before his disability began he had found that he was not making his letters correctly and could not go fast enough for the work. He had to give up his position in about ten weeks after the occurrence of the cramp.

Physical examination was in other respects negative, except for an exaggeration of the deep reflexes.

This summary analysis must be compared with and interpreted in the light of those I have published in the Journal of Psychology and Neurology of Leipsic, 1912 (Bul. 19). There, three cases of writer's cramp of different complexity are fully presented, and the method of management which led to their recovery is described, and the principles thereof are set forth. They were, shortly, as follows:

ILLUSTRATIVE CASES OF OCCUPATIONAL "NEUROSIS."

*Case I.*—A paymaster was obligated to sign over 200 checks a day. After an attack of "grippe" his signature had become a little uncertain, and one day it was refused by the bank. Dread of losing his position further augmented his difficulty; but he consulted me early, and one interview placed him in the way of cure after a few weeks' absence from work. His hardly legible scrawl at that time contrasts sharply with the bold and clear signature two and a half years later. The steps in the treatment were: (1) Discovery of the cause, an induced fear; (2) removal of the besetment; and (3) correction of the awkward position of the hands and the excessive rigidity of the muscles of the arm. This last was accomplished by gymnastics designed to give free movements and by the frequent practice of free writing in a smooth, large, round hand, a little at a time.

*Case II.*—A woman had always disliked correspondence, and in consequence wrote very rapidly. During a time of domestic stress and anxiety on account of sickness, she was particularly impatient of the Christmas letters she had to write, and the cramp ensued. When I saw her, two and a half years later, however, there was no marked fear affect nor insistent desire to recover. But, in about three months, she gradually became able to write a normal hand without difficulty, as a result of graduated exercises made possible by freeing her mind from the besetment which produced the cramp when she attempted to write.

*Case III.*—Singer's cramp and pen paralysis in a psychasthenic young woman. At the age of 19 she received, after a concert, an anonymous letter which created shame when she sang in public thereafter. Her anxiety to be a successful singer, in conflict with this shame, produced a pharyngeal cramp, in consequence of which she had to give up singing for some years. Fear of laryngeal tuberculosis also played a part in this. Some years later a period of stress and poor health, combined with hyperconscientious efforts to do full credit to a difficult clerical post, led to writer's cramp. She was sent to me two years later, after various unsuccessful medical and orthopedic attempts had been made to cure her. She was in despair at being unable to write, as she had to make her living; and although she had persevered, she could not face an audience in solo, so that her earnings as a singer were insignificant. After six months of most arduous treatment she became able to write perfectly with both left and right hands, and the singing cramp was greatly improved. She has already taken a clerical position, and is about to take a choir position once more.

In this case the chief difficulty was to overcome the patient's false belief in a physical disease, first in the hand, then in the shoulder; later, in the constitutional state due to a hypothetical tapeworm, and, finally, belief in a cerebral defect of neurasthenic type not psychological in mechanism. Eventual success in removing these beliefs led to the fruitful practice of gymnastic and caligraphic exercises, which before that had only aggravated her disability. (The patient has since relapsed as to writing with the right hand, but has learned to use the typewriter with it; has sung regularly for over a year.)

These cases clearly show the prepossession of the patient by *the idea of disability*. This notion breeds anxiety regarding capacity to perform the task required, writing or singing or what not. The nervous erethism of the anxious state is concentrated upon the apparatus required for performing the desired act of occupation. In this way, there is induced the state known to golfers as "pressing," and seen familiarly in the stammering movements of those who are apprehensive under unusual mental stress in the performance of an act.

Physiologically there occurs an involuntary innervation, lacking in proper co-ordination, mainly of the muscles about to perform the desired act. Instead of being in a state of tonic relaxation, the normal psysiological one, they are already beginning to contract before the voluntary inception of the professional act. Both agonists and antagonists may contract irrespectively of one another.

The condition is quasi volitional, as it is contingent upon the mental processes preliminary to the occupational act. Its incoherence is due to emotion, and the emotion—fear—is due to the idea that the act cannot be performed. This idea has arisen in various ways, as shown by the cases.

### CRAMP NEUROSIS IS A TIC.

When occupational neurosis is dyskinetic in type, as in the cases cited, the cause of the disability is in reality the pre-emption of the muscles required in the act by a tonic tic. It is ideational in origin, and conforms in every way to the well known definition of Janet into which I need not enter here, as I discuss it in the memoir cited and in the current number of the *Journal of Abnormal Psychology* (September, 1912).

But when there is no question of dyskinesis in an occupational psychosis, the mechanism is essentially the same. For tic is merely a motor obsession contingent upon the idea of certain situations within or without the body. Pure besetment without motor expression is essentially the same in a psychological sense, as so clearly shown by Janet. Neither of these manifestations is ever unaccompanied by phobia or Angst. So much so that some have regarded anxiety as the genetic factor of obsessions. The clinical evidence that this is not so is abundant. That it is the notion of the situation in the patient's mind which is the determinant of the emotional reaction of his body, is shown by a series of analyses of children made by the writer before the American Psychopathological Association (May, 1912), as well as in a theoretical discussion with casuistical illustration.

It is this state of obsession, expressing itself in the fear of one's occupation, which has so unfortunately received the name of neurosis; for the name leads to the idea of therapeutic intervention toward the lower neurones, such as their stimulation by strychnine and other drugs believed to be tonic in effect; such as by giving douches to arouse the nervous system; by attempts to improve nutrition; through massage, exercise, more nourishing food; by the imposition of rest, on the supposition that there is exhaustion of the nervous system; by prescription of change of scene, under the idea that the patient's balance will be thereby restored. These measures, excellent enough when indicated, are quite beside the mark in the affection we are considering.

The essential preliminary to treatment is the getting rid of this idea. But before this is done, the patient must be thoroughly convinced of the fact that it is this idea that produces the disability, and that his previous belief that the disability is of physical origin, whether of muscle, nerve, or brain, is utterly erroneous. In other words, he must clearly comprehend the psycho-dynamic genesis of his condition.

The second step is for him to reacquire control of the psychomotor mechanism required in his occupational acts. This can only be attained by practice. In proportion to the length of time the perverted kinesis has obtained, these efforts to rectify it are difficult and prolonged. Thus the naval paymaster, who has been incapacitated only a few weeks, was restored in less than a month, after one interview. The woman who had practically ceased writing for two and a half years required four treatments and about four months of practice before she could again write freely. The young woman with multiple professional cramps required six months of the most arduous discipline and frequent consultations before she could again write with the right hand and sing without cramp of the throat. In the cases of numerous telegraphers I have studied, no treatment at all was undertaken, because in part of the long standing of their condition; but mainly because the tiring work of a telegrapher does not leave enough energy for the arduous discipline required for re-educating the mechanism of the professional act so complex, delicate, and exacting as the use of the Morse key.

Theoretically, it is clear, and, practically, my cases show, that there is only one direct therapeutic means for them. That may be summed up as psychotherapy.

Of course, the effort demanded in a psychotherapeutic education requires for its best fruit a well nourished nervous system; and the patient's hygiene must therefore be rectified, when defective; but that is only a help, and not a cure.

The same consideration applies to prophylaxis. It is of course desirable that workers should be well nourished, and that fatigue should be minimized; but this, in itself, will not prevent occupational neurosis, if the ideational seeds are not prevented from germinating in the minds of the workers.

